# Hydralazine-Induced Dual Antineutrophil Cytoplasmic Antibody Positive Vasculitis and Nephritis

**DOI:** 10.7759/cureus.8911

**Published:** 2020-06-29

**Authors:** Sandeep A Padala, Vidya M Medepalli, Azeem Mohammed, Stanley Nahman

**Affiliations:** 1 Nephrology, Augusta University Medical College of Georgia, Augusta, USA; 2 Medicine, Augusta University Medical College of Georgia, Augusta, USA

**Keywords:** hydralazine, drug-induced lupus (dil), antineutrophil cytoplasmic antibody (anca), antineutrophil cytoplasmic antibody (anca) associated vasculitis (aav), pauci-immune glomerulonephritis (gn), acute kidney injury (aki), dual anca aav and dil, hydralazine induced dual aav and dil

## Abstract

Drug-induced autoimmunity occurs when exposure to a causative agent leads to serologic or clinical autoimmune responses. Syndromes that may be associated with drug-induced autoimmunity include antineutrophil cytoplasmic antibody (ANCA) associated vasculitis (AAV) and drug-induced lupus (DIL). When drug-induced autoimmunity involves the kidney, histological patterns of injury include pauci-immune glomerulonephritis (GN), which occurs with AAV, and immune complex-mediated GN, which is associated with DIL. We present a case of hydralazine-induced dual ANCA-positive vasculitis and nephritis.

## Introduction

Hydralazine, an anti-hypertensive arterial vasodilator, was one of the first and remains a common cause of autoimmunity. In cases of hydralazine-associated autoimmune disease, renal involvement is more common with antineutrophil cytoplasmic antibody (ANCA) associated vasculitis (AAV). Drug-induced lupus (DIL) rarely involves the kidney, but the two syndromes may have similar clinical presentations, making an assessment for ANCA important in the evaluation [1,2]. The presence or absence of ANCAs is one of the most helpful clues; ANCAs are positive with AAV and generally not seen with immune complex glomerulonephritis (GN)/DIL [3]. Herein, we present a unique case of DIL nephritis with serologic and histologic features of both diseases.

## Case presentation

A 76-year-old Caucasian female was transferred to our institution for the evaluation of non-oliguric acute kidney injury (AKI) on chronic kidney disease (CKD). Her medical history included hypertension, type 2 diabetes mellitus, stage 3 CKD due to diabetic nephropathy (baseline serum creatinine of 1.5-2.0 mg/dL), coronary artery disease status post-multiple stents, peripheral arterial disease, and chronic diastolic heart failure. She denied any history of autoimmune disease or alopecia, photosensitive rash, oral ulcers or paresthesias, or family history of autoimmune disease. Home medications included amlodipine 5 mg daily, atenolol 50 mg daily, hydralazine 100 mg every eight hours, isosorbide mononitrate 60 mg daily, losartan 100 mg daily, aspirin 81 mg daily, clopidogrel 75 mg daily, and atorvastatin 10 mg daily. The patient was initially admitted to the outside facility for acute hypoxemic respiratory failure and a urinary infection, which was treated with ceftriaxone. There was a two-month history of fatigue, arthralgias, and recurrent sinus infections. A CT angiogram ruled out pulmonary embolism, but the serum creatinine rose from 2.0 to 5.2 mg/dL within 24 hours. The etiology of the AKI was thought to be contrast nephropathy. Despite supportive care, the renal function worsened and she was transferred to our institution for further evaluation.

Upon admission, vitals included a temperature of 37.4°C, heart rate of 69 beats per minute, blood pressure of 172/69 mmHg, respiratory rate of 18 breaths per minute, and O_2_ saturation of 99% on room air. On physical examination, she was comfortable, with moist oral mucosa. The lungs were clear to auscultation, and the cardiovascular examination revealed a systolic murmur without jugular venous distension. There was 1+ bilateral lower extremity edema. There were no rashes or other skin lesions. Nephrology was consulted for non-oliguric AKI with worsening renal indices. Urine microscopy showed many red blood cells but no acanthocytes or casts. Renal ultrasound revealed 12-cm kidneys bilaterally with cortical thinning without mass or obstruction. The initial working diagnosis was AKI on CKD secondary to contrast-induced nephropathy or acute interstitial nephritis following ceftriaxone exposure, atheroembolic disease, and systemic vasculitis. As shown in Table 1, there was an increase in antinuclear antibody (ANA), elevated double-stranded DNA (ds-DNA), ANCAs, and a mildly depressed C3, raising the concern for possible hydralazine-induced AAV and DIL. Hydralazine was stopped, and high-dose steroids were initiated pending a renal biopsy. Due to volume overload and worsening renal indices, renal replacement therapy was also initiated.

**Table 1:** Laboratory Data CRP, C reactive antibody; ANA, antinuclear antibodies; RF, rheumatoid factor; C3, complement component 3; C4, complement component 4; HIV, human immunodeficiency virus; dsDNA, double-stranded DNA antibody; ANCA PR3, antineutrophil cytoplasmic antibody proteinase 3; ANCA MPO, antineutrophil cytoplasmic antibody myeloperoxidase; SPEP, serum protein electrophoresis; SIFE, serum immunofixation; SFLC ratio, serum free light chains ratio (Kappa/Lambda)

White blood count (4.5-11 × 10^3^ cells/mm^3^)	5.1
Red blood count (4.2-5.5 million/mm^3^)	2.5
Hemoglobin (12-16 g/dL)	7.2
Hematocrit (37-47%)	21.1
Platelet (150,000 to 400,000/mm^3^)	206
Sodium (135-145 mEq/L)	135
Potassium (3.5-5.5 mEq/L)	4.3
Chloride (99-109 mEq/L)	102
Bicarbonate (20-31 mEq/L)	24
Blood urea nitrogen (9-23 mg/dL)	41
Creatinine (0.6-1.6 mg/dL)	5.22
Glucose (74-106 mg/dL)	114
Calcium (8.7-10.4 mg/dL)	9.0
Albumin (3.2-4.8 g/dL)	3.2
Total bilirubin (0.3-1.2 mg/dL)	0.4
Phosphorus (2.4-5.1 mg/dL)	5.1
Magnesium (1.3-2.7 mg/dL)	2.0
CRP (0-0.5 mg/dL)	3.125
ANA screen	Positive
ANA titer	≥1:640
RF (0-14 IU/mL)	24
C3 (90-170 mg/dL)	77
C4 (12-36 mg/dL)	12
Cryoglobulin	Negative
Hepatitis screen	Negative
HIV	Negative
dsDNA (<30 IU/mL)	115
ANCA PR3 antibody (<0.4 U)	3.5
ANCA MPO antibody (<0.4 U)	7.6
Anti-histone antibodies (<1 U)	5.3
SPEP	No monoclonal immunoglobulins detected
SIFE	No monoclonal immunoglobulins detected
SFLC ratio (0.26-1.65)	0.7

**Figure 1:**
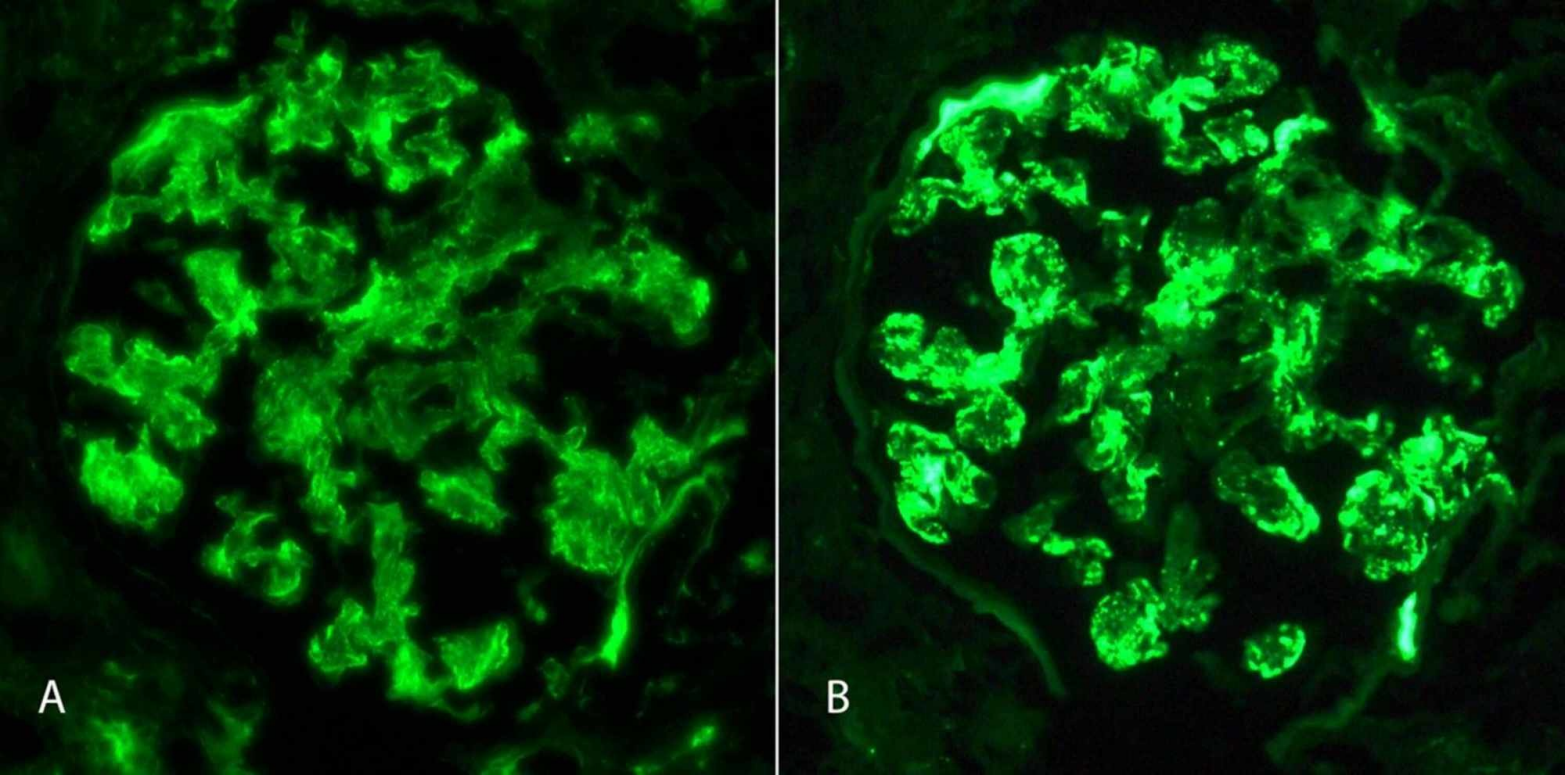
Immunofluorescence Microscopy Ten glomeruli were available for evaluation, of which none were globally sclerosed. There was granular mesangial and capillary wall staining with antisera specific for IgG (segmental trace), IgA (trace), IgM (4+), complement factor C3 (4+), kappa light chain (1+), and lambda light chain (3-4+). No staining was seen with C1q and fibrin. No diagnostic extraglomerular staining was seen with antisera specific for IgG. Ig, immunoglobulin

**Figure 2:**
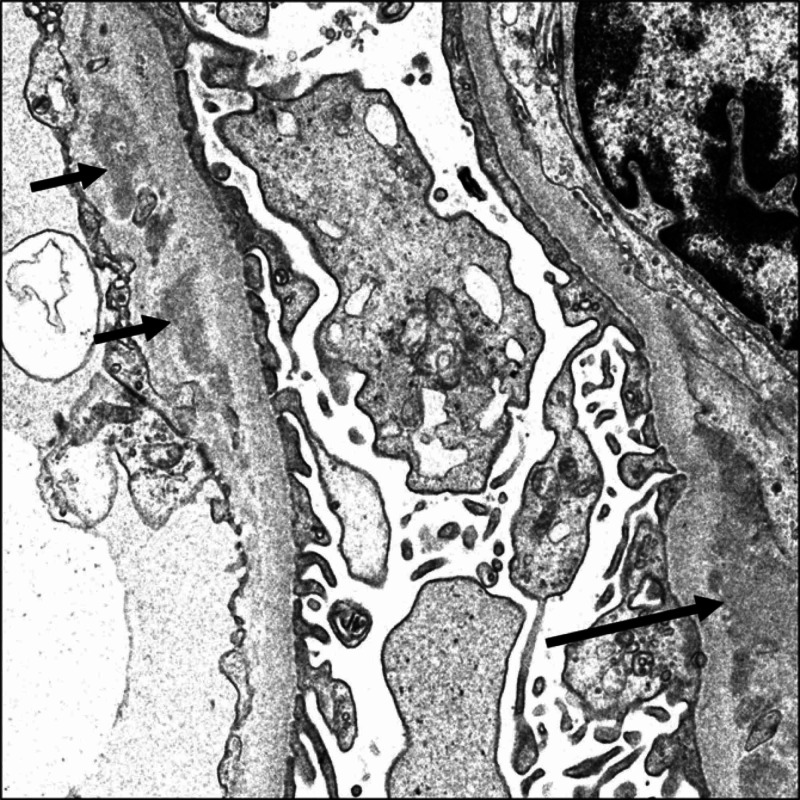
Electron Microscopy Out of two glomeruli, none revealed global glomerulosclerosis. The glomeruli revealed mild mesangial matrix expansion. The tubulointerstitial compartment revealed mild acute tubular injury. One small interlobular artery cross-section with mild intimal fibroelastosis was also seen. Ultrastructural examination demonstrated peripheral capillary loops segmental thickening of the lamina densa associated with segmental remodeling of some of the capillary loops. There was widespread effacement of the overlying foot processes with associated moderate microvillus transformation of the podocyte cytoplasm. Mesangial, paramesangial, and subendothelial immune complex-type electron-dense deposits were noted. The immune complex-type electron-dense deposits did not reveal any characteristic and diagnostic substructure or organoid pattern. No endothelial cytoplasmic tubuloreticular inclusions were identified. Light microscopy: Out of three glomeruli per level of section for evaluation, none were globally sclerosed. The glomeruli revealed mild mesangial matrix expansion with associated mild mesangial cells hypercellularity and segmental mild endocapillary hypercellularity. One segmental cellular crescent was noted. No fibrinoid necrosis or segmental scarring was identified. The tubulointerstitial compartment revealed diffuse acute tubular injury, interstitial edema, foci of mononuclear cells with neutrophilic component interstitial infiltration, and red blood cell cast formation. There were mild interstitial fibrosis and tubular atrophy. The few sampled arterioles were unremarkable, and the arteries were not sampled.

The biopsy (Figures 1, 2) revealed immunoglobulin (Ig) M dominant immune complex-mediated focal proliferative GN. Light microscopy showed one segmental crescent, mild mesangial hypercellularity, acute tubular injury, interstitial edema, foci of mononuclear cells with neutrophilic interstitial infiltration, and red cell casts. Immunofluorescence showed granular mesangial and capillary wall staining for IgM (4+), C3 (4+), lambda light chain (3-4+), trace IgA, and segmental trace IgG. Electron microscopy showed definitive immune complex deposits in the mesangial, para-mesangial, and subendothelial compartments.

Based on the biopsy findings, AKI was presumed to be from hydralazine-induced dual ANCA AAV and DIL. The patient was offered cyclophosphamide or rituximab in addition to continuing prednisone, but she declined an escalation of immunosuppression. The renal function did not recover, and she became dialysis-dependent.

## Discussion

Our patient most likely demonstrated AKI from hydralazine-induced dual ANCA AAV and DIL. This hypothesis is supported by the renal biopsy findings and clinical serologies. ANCA AAV and DIL have each been reported with hydralazine, but the present case shows the unusual occurrence of the simultaneous expression of both conditions in the same patient.

Hydralazine is often implicated as a causative factor in DIL, but it rarely causes AAV. DIL is characterized by fever, polyarthralgias, and pleuritic pain [1,2], whereas AAV is more commonly associated with leukocytoclastic vasculitis, pulmonary infiltrates, elevated ANCA levels, and pauci-immune GN on renal biopsy [1,2]. Our patient exhibited no clear clinical signs of DIL or AAV, but the serologic evaluation suggested the possibility of both diagnoses. A renal biopsy confirmed the hypothesis. While the two processes have distinct histopathologic findings, ANCAs are an important means of differentiating the two before biopsy [3]. It has been shown that both DIL and ANCA AAV may represent a spectrum of the same disease [2].

The serologic findings in DIL typically include a positive ANA, anti-histone antibody, low complements [4,5], and variable anti-dsDNA (double-stranded DNA antibody) antibody levels. Renal involvement in DIL is rare, with only a few cases described in the literature. Shapiro et al. described a case of DIL due to hydralazine, with the biopsy demonstrating immune complex-mediated GN [6]. Sheikh et al. reported a case of crescentic GN in a patient with DIL due to procainamide [7].

The serologic findings in drug-induced AAV include a positive ANA, ANCA, and variable anti-dsDNA and anti-histone antibody and complement levels [8]. Renal involvement is a common occurrence in cases of AAV due to hydralazine and is typically seen early in the clinical course. Renal biopsy in these cases demonstrates pauci-immune GN [5,9].

A case series of ANCA-associated GN due to hydralazine by Kumar et al. discusses the overlapping clinicopathological features of DIL and AAV and proposes that hydralazine-associated vasculitis and lupus are related disease processes that exist on a continuum [2]. The risk-benefit ratio and implications of continuing therapy should be considered in the case of a positive or rising ANA titer [2]. Espinosa et al. also described the simultaneous presentation of DIL and ANCA AAV secondary to hydralazine in a sarcoid patient [10]. Our case supports the co-expression of these diseases in patients treated with hydralazine. The presence of end-organ damage implies poor prognosis with high morbidity and mortality [11].

It has been reported that the overlapping presentation of both DIL and ANCA AAV have positive ANA, anti-histone antibodies, ds-DNA, elevated ANA, and ANCA titers [11], as evidenced in our case. The presence of anti-histone antibodies in a patient on hydralazine should raise the suspicion of DIL [12,13]. The risk of hydralazine-induced DIL is high in females, slow hepatic acetylators, human leukocyte antigen-DR4 (HLA-DR4 genotype), doses more than 200 mg/day, and individuals with the null gene for complement system protein C4 [14,15].

When hydralazine is suspected to be the cause, there is a temporal correlation with improvement in the renal indices after the medication is stopped [12]. The possibility of hydralazine-induced vasculitis should be suspected in any patient presenting with pulmonary-renal syndromes [3]. Our case is unique since hydralazine-induced lupus nephritis is a rare occurrence, with concurrent AAV causing further renal damage. The most important step in the management of this rare disease is discontinuation of the medication. If there is no improvement and or worsening of the renal indices, then treatment with plasmapheresis, cyclophosphamide, or rituximab should be considered [16]. Our patient was on hydralazine 100 mg every eight hours for more than five years, which could have increased the risk of AAV and DIL.

## Conclusions

Our case represents the overlapping features of both AAV and DIL. Although the patient had a clinical and laboratory presentation consistent with ANCA vasculitis, renal biopsy showed immune complex deposits and was suggestive of DIL nephritis. Hydralazine-associated vasculitis and lupus are related disease processes that exist on a continuum. We would like to point out that since hydralazine is known to cause DIL and ANCA vasculitis, is it imperative to get a baseline ANA titers and monitor the titers periodically if prolonged therapy is indicated, even if the patient is asymptomatic. Further studies regarding monitoring the titers may help us guide in a better direction and avoid the risk of drug reactions. The risk-benefit ratio and implications of continuing therapy should be carefully considered in the case of a positive or rising ANA titer. Increased awareness and a high degree of clinical suspicion can help the clinicians diagnose this rare condition caused by the common medication, thus improving overall morbidity and mortality.
